# 

**Published:** 1918-07-27

**Authors:** 


July 27th, 1918.] [Price Twopence

### The Hospital

The Workers' Newspaper of Administrative Medicine and Institutional Life, Administration, National Insurance and Health.

CONTENTS.

The Ministry of Health... ... ... ... ... 357

Medical Women in the Army... ... ... ... 358

The Great War ... ... ... ... ... ... 363

XI. Personal Cleanliness.

Temporary Artificial Legs (Illustrated) ... ... 365
IV. Details of Organisation.

The History of the Order of St. John ... ... 367
Progressive Developments from 1090 Onwards.
Value of First Aid in the Management of Mental Cases and Hospitals.—Part II.

Hospital and Institutional News ... ... ... 359
Where is Mr. Hodge, Minister of Pensions?—What are M.P.s About—Asleep?—Canada and the U-boat Devilry—Why Does Mr. Lloyd George Nod?—Professional Classes' Maternity Home—Lectures to Patients—A Secretary as a Patient—The Blessings Book—The Control of Auxiliary Hospitals—Discharged Soldiers and Clare Hall Sanatorium—The Night-dwellers in the City—The Duchess of Norfolk at the Jessop Hospital—The Talkative Nurse—A Matron on Military English—Who Pays the Postman?—A Lay View of the Nurse's Training—Hospital Work in New Zealand—Jews and Post-mortems—This Week's Drug Market.

The London Hospital Nurses ... ... ... ... 375
A Race Between Statesmanship and Economics.

The Making of Profit and the British Nurse ... 370
The Argument and the Moral.

The Empire Aroused ... ... ... ... ... ... 369

Australian Troops' Bravery.

The Leicester Royal Infirmary ... ... ... 376
Interesting Points from Half-yearly Meeting.

The Matrons', Sisters', and Women's Departments 371
The Call to Strengthen the Nursing Profession.
Round the Hospitals.
Intercession: Why the Doctor and the Nurse, *not* the Archbishops, are Right—What is the Archbishops' Answer?—The Kitchen Mistress, Edinburgh—Why "Overseas" is Preferred to "Colonial"—The Talkative Nurse—The Nursing Sister and Her Security—40,000 Fully Qualified for the College.

Parliament and the Profession ... ... ... ... 377

The Woman Workers' World ... ... ... ... 377

The Institutional Worker ... ... ... ... ... I
The Steward's Department of a Large Hospital.

